# Testing the impact of effective population size on speciation rates – a negative correlation or lack thereof in lichenized fungi

**DOI:** 10.1038/s41598-018-24120-9

**Published:** 2018-04-10

**Authors:** Jen-Pan Huang, Steven D. Leavitt, H. Thorsten Lumbsch

**Affiliations:** 1 Integrative Research Center, The Field Museum, Chicago, USA; 2 Department of Biology & M. L. Bean Museum, Brigham Young University, Provo, USA

## Abstract

The effect of microevolutionary processes on macroevolutionary patterns, and *vice versa*, is an important but under-investigated question. Here we present an integrative population genetic and phylogenetic study using molecular sequence data from three lichen-forming fungal lineages to empirically test the potential correlation between effective population size – approximated by the parameter *θ* – and estimated speciation rates using a phylogenetic tree (*λ*). A negative association between *θ* and *λ* was supported in one lineage of lichen-forming fungi, *Melanelixia* (Parmeliaceae), while no significant relationships was found for two other genera within the same family, *Melanohalea* and *Xanthoparmelia*. We discuss the significance of our results and the importance of considering microevolutionary processes when studying macroevolutionary patterns.

## Introduction

Whether macroevolutionary patterns can be explained by microevolutionary processes, and *vice versa*, is a critical, unsettled question in evolutionary biology^1–4^. Many studies have been devoted to bridge microevolutionary and macroevolutionary patterns^2,3^. For example, speciation has been hypothesized to be a driving force accelerating molecular evolution^4^ (but see^5^), and a positive correlation between speciation rate and the rate of molecular evolution between lineages have been reported at both adaptive and neutral genomic regions^2^. In comparison to investigations focusing on the correlation between the rate of speciation and molecular evolution among species, the impact of effective population size^1^ has seldom been empirically investigated within a macroevolutionary framework^6,7^. However, the potential effect of differences in effective populations sizes on variation in speciation rates across evolutionary lineages has been commonly discussed^6,8^.

Genetically structured populations can be in initial phases of becoming different species, and the rate to generate structured populations, which can be affected by size of the populations, may relate to the rate of speciation^9,10^. Predictions for positive, negative, and no correlation between effective population size and speciation rate have been proposed. For example, small effective population sizes may facilitate rapid genetic differentiation between subdividing populations^1^. However, based on the neutral theory, the only demographic parameter that may affect the rate of organismal diversification is generation time^11^, if speciation results from the accumulation of fixed mutations between diverging taxa. Furthermore, if the diversification process is selection-driven, a larger effective population size may confer to a higher speciation rate. Empirical studies that specifically test these predictions and discuss the effects of effective population size on speciation rate are still scarce^7,9–11^.

Multiple factors can affect intraspecific genetic diversity, e.g., *θ*, which is often used to approximate effective population size, and the main determinant may vary among evolutionary lineages^6^. Note that *θ* is a function of effective population size (N) and the per-generation mutation rate (µ) (*θ* = 4Nµ). While µ tends to vary by less than an order of magnitude in real data, N varies by several orders of magnitude^12^. Therefore, estimated *θ* values can be used as proxies for population size. However, directly comparing and contrasting results among studies is often unrealistic and potentially problematic due to differences in methods proposed to estimate speciation rate from a phylogenetic tree (e.g., Pagel’s *λ* versus Magallon & Sanderson’s *λ*)^13,14^.

Lichenized fungi represent a highly-diversified group, for which species diversity has generally been under-estimated^15,16^. A broad sampling of DNA sequence data from previous molecular species delimitation studies allow us to estimate intraspecific genetic diversity (*θ*) as an approximation for effective population size, in addition to phylogenetic reconstruction and the estimation of speciation rate (*λ*). We used sequence data from three genera in the hyperdiverse family Parmeliaceae to test the correlation between *θ* and *λ* – *Xanthoparmelia*, *Melanelixia*, and *Melanohalea*. These genera share a most recent common ancestor at ca. 68 Myr, and each lineage has a Neogene-dominated diversification history^17^. *Melanelixia* and *Melanohalea* have moderate species numbers (<30), and most species are represented by DNA sequence data and dense, intraspecific specimen sampling. In contrast, *Xanthoparmelia* is highly diverse, with over 800 species^18^, and dense specimen sampling is limited to a small number of well-sampled clades. Differences in species-level circumscriptions among taxonomists may have significant effect on the estimations of population genetic parameters, and our sampling strategy attempted to minimize the effect of such inconsistency on assessing the correlation between *θ* and *λ*. Specifically, these three genera were selected because their taxonomic status has been recently revised using similar empirical species delimitation methods based on multi-locus sequence data^19,20^. Our study question concerns local *θ*, which are the basic units of evolution that contain randomly mating individuals, and for most sampled species, specimens were collected from multiple, geographically distinct populations^19,20^.

In this study, we evaluate the association between *θ* and *λ* using recently revised species-level taxonomy for three genera in Parmeliaceae, in addition to applying a molecular species delimitation approach (GMYC) that has been shown to effectively delimit local populations as independent evolving lineages/species should genetic structure exists^21^. *θ* values were estimated for different species delineation scenarios based on the number of segregating sites per site, and *λ* values were estimated using Magallon and Sanderson’s method^14^. Below we report the results and discuss implications of our study.

## Results

The quality of the internal transcribed spacer region (ITS) multiple sequence alignment, a maximum likelihood (ML) reconstruction of the ITS phylogeny, and a chronogram based on the ITS ML phylogeny can be found in the supplementary data (Figs S1, S2, and S3). The total length of the ITS sequences after alignment and quality filtering was 565 base pairs. The ITS region does not encode protein sequence and is presumably evolving neutrally. Therefore, variation in the ITS region, and thus the estimated *θ* value, can be assumed to be an appropriate proxy for effective population size because it is not expected to show large variation in mutation rate across lineages.

A total of 74 species were delineated based on the results of previous multi-locus species delineation studies^19,20^ – 22 species in *Xanthoparmelia*, 18 in *Melanelixia*, and 26 in *Melanohalea*. In addition to previous, multi-locus species circumscriptions, for this study species delineations were also determined using a Bayesian Generalized Mixed Yule Coalescent (bGMYC)^21^ approach based on sequence data from the ITS region. A total of 437 species were delimited using bGMYC of the ITS topology (Fig. S4), with 102 in *Xanthoparmelia*, 110 in *Melanelixia*, and 192 in *Melanohalea*. The estimated *λ* varied among lineages, with species from the *Xanthoparmelia* lineage having the highest values (Figs S5 and S6). Some species from the *Melanelixia* lineage also exhibited high *λ* values but only under the bGMYC species delimitation scenario (Fig. S6).

**Figure 1:**
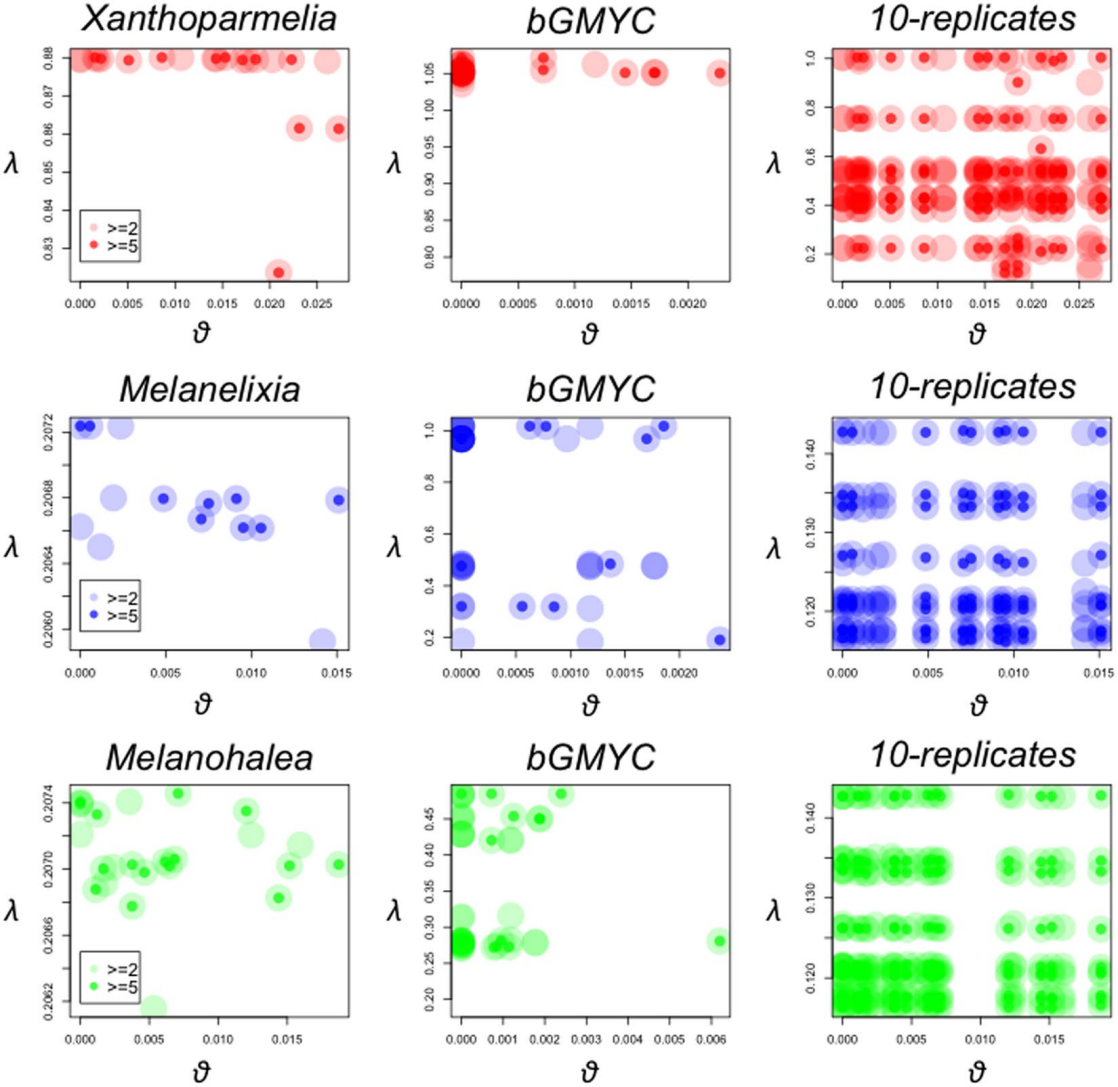
Scatter plots of estimated effective population size parameter (*θ*, estimated based on the number of segregating sites per site; *θ* = 4 Nµ) versus estimated speciation rate (*λ*, estimated based on reference^14^ and the programs BAMM^23^ and BAMMtools^33^) for tip taxon from three lineages of lichenized fungi. Left panels: results from species delineated based on previous species delimitation results. Middle panels: results from species delineated based on bGMYC species delimitation. Right panels: results from ten replicates using ten randomly chosen post burnin MrBayes trees and species delineated based on previous species delimitation.

The distributions of estimated *θ* from different species overlapped among the three lineages (Figs S7 and S8). The estimated *θ*, inferred based on the number of segregating sites per site, was smaller when species were delineated via bGMYC than those based on results from previous multi-locus species delineation analyses (Figs S7 and S8). Statistically significant correlation between *θ* and *λ* after controlling for phylogenetic relatedness (i.e., phylogenetic independent contrast; PIC) was found in the *Melanelixia* lineage, where species were delineated based on multi-locus sequence data^19^ (*P* = 0.0002832 and R^2^ = 0.8885 for comparison that only included species that have at least five sampled individuals [> = 5]; Fig. 1). All the other comparisons revealed insignificant results.

In the assessment of the impact of uncertainty in phylogenetic reconstruction and its effect on estimated divergence times and speciation rates, only a single significant correlation between *θ* and *λ* was detected from ten randomly sampled post-burnin trees. The significant result was found in the *Melanelixia* lineage when species were delineated based on previous study^19^ and for comparison that only included species that have at least five sampled individuals. All the other comparisons revealed insignificant results (Fig. 1). Note that an independent bGMYC analysis was not implemented for each of the ten replications.

## Discussion

Our study addressing the impact of effective population sizes on speciation rates in lichenized fungal lineages revealed a correlation between *θ* and *λ* values in one of the three empirical cases investigated here. However, our results varied based on how species were delineated and by the number of individuals per species that were sampled. We emphasize that our use of the term ‘species’, as it relates to the different species delimitation scenarios, refers to putative evolutionarily independent species-level lineages. The two different species delimitation scenarios provide strikingly different perspectives into delineations of species-level lineage in these symbiotic fungi – 74 putative species-level lineages using and integrative approach vs. 437 putative species-level lineages based on a bGMYC analysis of the ITS multiple sequence alignment alone. No significant correction between *θ* and *λ* values was found when using species delineations based on the bGMYC analyses. In the single case supporting a significant correlation between *θ* and *λ* – *Melanelixia* species delimited using an integrative approach (from a previous study^19^) and with each taxon represented by at least five samples to estimate the *θ* value – a smaller *θ* was associated with a higher *λ*. This result implies that mechanisms that generate microevolutionary diversity might also help shape macroevolutionary patterns in some cases. However, the limited scope of the number of lineages analyzed in this study and a lack of correlation between *θ* and *λ* for the remaining clades limits our ability to make broad, generalizable inferences. Low *θ* caused for example by founder events has been hypothesized to accelerate population subdivision and thus may lead to high speciation rate. A high speciation rate may in return cause low genetic diversity, e.g., *θ*, within species and high genetic divergence between species^4,6^.

Lineage-specific diversification patterns, where some evolutionary lineages accumulate more species than the others given the same time^22^, has been a main focus in macroevolutionary studies. Models have been developed not only to detect lineage-specific diversification rate^22,23^, but also to test if such pattern is associated with key innovations and geological/climatic events^23,24^. However, without statistically rejecting the effect of stochastic processes that may also lead to variation in diversification rate across evolutionary lineages, the test of trait-dependent speciation and discussion on adaptive radiation seem *ad hoc*^24^. Unlike deterministic mechanisms, such as key innovations and adaptive evolution, that have been the foci of macroevolutionary studies, the equally important stochastic mechanisms, e.g. demographic history, have rarely been incorporated in macroevolutionary models. Our results suggest that stochastic processes might result in differences in speciation rates among lineages in lichenized fungi. Therefore, we caution against testing and inferring trait-dependent diversification patterns without rejecting the effect from stochastic process.

In two of the three empirical cases investigated here, an association between *θ* and *λ* could not be found (Fig. 1), implying that other processes governing the transition between micro- and macroevolutionary diversity patterns likely exist^9^. Mechanisms that can initiate population subdivision may not always result in speciation events^9^. For example, populations structured by geographic isolation may not acquire ecological differences or reproductive barriers, and a panmictic population may result if secondary contact occurs. Similarly, ecotypes formed by adaptation into different micro-habitats may not become distinct species if the selective pressure is weak^10^.

Alternatively, a lack of correlation between *θ* and *λ* may, however, simply indicate that factors other than *θ* may have a larger impact on *λ*. For example, it has been shown that generation time is highly correlated to the number of species in midges^11^. It is indeed unreasonable to assume that all the species in the three lichenized fungal lineages investigated here have the same generation time. These fungal lineages exhibit different life styles, e.g., asexual versus sexual, and live in a wide range of habitat types, e.g., arid versus temperate regions. Crucial life history information is unfortunately incomplete in our study system, and the impact of generation time on *λ* cannot be properly addressed here. Additionally, symbiotic relationships with different algal lineages between fungal lineages may have a large effect on adaptive evolution^18^. The effect of population size, or stochastic process on speciation rate can be diminished when speciation is predominantly selective driven. Furthermore, how many lineages and samples per species are needed to confidently detect significant/non-significant correlation between *θ* and *λ* is beyond the scope of our study. The possibility that our non-significant results are due to the lack of statistic power cannot be excluded.

In addition to the lack of complete life history data and power analysis mentioned previously, numerous assumptions are inherent in species delimitation using gene trees^21^, the use of *θ* to represent effective population size^6^ (especially the *θ* value was estimated from only one genetic marker^6^), and the estimation of *λ* using the BAMM model^14,23^. How these assumptions may affect our results is not clear. The hypothesis addressed in our study concerns intraspecific, or local *θ*, where individuals within each population/species mate randomly. Should within-species genetic structure exists (e.g., geographically or ecologically structured forms/populations), the estimated *θ* can be inflated and lead to biased results. Nevertheless, when a species delimitation method that has been shown able to delimit populations, or fine scale genetic structure, effectively was applied to our dataset (i.e., GMYC), we obtained results consistent with those from integrative taxonomic circumscriptions.

The GMYC approach consistently delimited more species in our study than previous circumscriptions based on multi-locus sequence data and resulted in fewer individuals per species. The GMYC approach may not only lead to a smaller *θ* value and a higher *λ*, but also further reduce the statistical power to detect correlation. An additional assumption made in this study is that the measurement of current genetic diversity can be related to the estimation of speciation rate from a phylogenetic tree. Although the BAMM analyses can be used to estimate speciation rate at each tip, the estimated rate is integrated over the past million years, depending on where in the phylogeny the rate shift was identified by the model. Although our novel, innovative approach provides important insight into the connection between microevolutionary and macroevolutionary processes, these results should be interpreted with caution, and future studies are needed to fully investigate whether the patterns reported in the lichen system are robust. Specifically, we did not reject the hypothesis that a small *θ* may lead to a larger *λ*. Instead, our results indicate that with the currently available data, we can only detect a significant negative correlation between *θ* and *λ* in one lichen lineage.

Our study demonstrates that diverse evolutionary lineages of lichenized fungi are great candidates to bridge micro- and macroevolutionary studies. Due to the prevalence of phenotypically cryptic species in many nominal lichenized fungal species, multiple samples per species are commonly sequenced, which enable both population genetic and phylogenetic approaches using the same system. This is a great advantage over phylogenetic studies that only sequence a single, or a limited number of samples per species, and population genetic studies, which often do not have a wide taxonomic sampling breadth. Future studies incorporating multi-locus and genome-scale data, as well as new models that account for population processes on speciation^9^, and life history data will help to understand not only how the genetic diversity was generated, but also maintained through evolutionary time in the natural system.

## Materials and Methods

A total of 1013 internal transcribed spacer region (ITS) sequences from *Xanthoparmelia* (n = 198), *Melanohalea* (489), *Melanelixia* (264), and their close relatives, *Montanelia* (60) and *Emodomelanelia* (2), were obtained from TreeBase (S11457 & S18577). A previous species delimitation and taxonomic study of *Melanelixia* and *Melanohalea* was based on sampling of worldwide populations, and approximately 20% of species in these two genera are represented by sequence data from specimens collected from multiple, geographically distinct populations^19^. Our sequence sampling of *Xanthoparmelia* species was largely based on populations in western North America (approximately 4% of the named species), with each species-level lineage represented by samples collected from multiple, geographically distinct populations^21^.

Sequences were aligned using MAFFT ver. 7.304^25^ and the quality of the alignment was calculated using Guidance 2^26^. A phylogenetic reconstruction of ITS was performed using a maximum likelihood (ML) method via phyML^27^, specifying an automatic sequence evolutionary model selection. The resulted ML phylogeny was then imported into PATHd8^28^ and converted to a chronogram with the root of the phylogeny specified as 68 MYA^17^.

Species delineations in this study were based on two different scenarios: (1) results from recent multi-locus species delineation studies^19,20^ and (2) a Bayesian Generalized Mixed Yule Coalescent (bGMYC^21^) approach based on ITS sequence data alone. The taxonomy of the brown parmelioid genera, which includes *Melanohalea* and *Melanelixia*, was recently revised^19^, reflecting empirical species delimitation studies. However, while species boundaries in *Xanthoparmelia* has also been circumscribed using empirical species delimitation analyses^20^, a formal taxonomic revision has not yet been proposed. We were fully aware of potential over-splitting species when applying the GMYC processes for species delimitation^29^. However, the rationale here was to investigate the inconsistency of our results. We followed the suggested procedure by Talavera and collegues^29^ for our bGMYC analysis.

The MCMC searches for bGMYC were launched for 5 $\times$ 10^5^ generations while thinning every 1 $\times$ 10^3^ generations and a burnin of the first 10% of samples. Species were delineated using a threshold of 0.05 from the bGMYC result, which yielded a conservative estimate of species number^21^. Species with multiple samples (delineated under both scenarios) were pruned using the drop.tip function in R package ape^30^. The edited species trees, where each delineated species contains only one representative sample, were saved in newick format.

The speciation, extinction, and net diversification rates of the three lichenized fungal genera were estimated based on the edited species trees and a Bayesian Analysis of Macroevolutionary Mixtures (BAMM ver. 2.5)^23^. A recent study highlighted potential limitations of using BAMM to study macroevolutionary dynamics^31^ (but see^32^). However, the estimated rate has been shown to correlate with the true rate using simulation^32^. In our study, we focused on the relative rate difference between species, and BAMM represented a suitable model for our study quest. We used the function setBAMMpriors from the R package BAMMtools^33^ to specify initial *λ*, extinction rate, and rate shift values. Sample fraction files that specified the proportion of missing species from the phylogenetic tree were provided for the BAMM analyses (available upon request). A total of 1 $\times$ 10^8^ MCMC searches were run for the BAMM analyses with samples stored every 1 $\times$ 10^4^ generations. After discarding the first 50% of samples as burnin, the remaining MCMC samples from the BAMM analyses were used to estimate *λ* values at tips of the trees via the getTipRates function.

To account for uncertainty in phylogenetic reconstruction and its effect on the estimated divergence times and speciation rates, we repeated the BAMM analyses ten times by using different trees as inputs. Specifically, a Bayesian phylogenetic reconstruction was performed using the program MrBayes (ver. 3. 2. 6)^34^. A GTR + I + G sequence evolution model (nst = 6 and rate = invgamma) was specified for the analysis. The analysis was launched for 1 $\times$ 10^8^ MCMC generations with a sampling frequency of every 100 generations. The split frequency decreased below 0.01 after generation 3 $\times$ 10^7^. The burnin period was thus set to 3 $\times$ 10^7^ generations. We randomly selected ten trees from the 70000 post burnin trees. The selected trees were converted into ultrametric trees with a root depth at 68 MYA using PATHd8. The converted ultrametric trees were then imported into BAMM for ten replicate analyses to estimate *λ* values at tips.

Sequences from multiple individuals of the same species (delineated under both scenarios) were used to calculate the number of segregating sites with a customized R script (indels ‘-’ and missing data ‘?’ were not counted as segregating sites). We subsequently used the function theta.s from the pegas package^35^ to estimate *θ* (*θ* = 4 Nµ) for different species based on the number of segregating sites. The value of *θ* represents the expected number of variable sites per site between two sequences randomly pulled from a sequence alignment. We estimated the *θ* values for different species with two minimum numbers of individual as thresholds – at least 2 (size 2) and 5 (size 5) individuals have to present in a species for that species to be included in the analyses. A linear regression to test for significant correlation between *θ* and *λ* for the three lichenized fungi lineages was statistically evaluated. We controlled for phylogenetic non-independence of *θ* and *λ* values by using the phylogenic independence contrast via the pic function implemented in ape^30^.

## Electronic supplementary material

Supporting information
