# Supplementary material for: Testing the impact of effective population size on speciation rates – a negative correlation or lack thereof in lichenized fungi
Source: Sci Rep. 2018 Apr 10;8:5729. doi: 10.1038/s41598-018-24120-9 (PMC5893563; doi:10.1038/s41598-018-24120-9)
Supplement: Supplementary file 1 — Supporting information [file 41598_2018_24120_MOESM1_ESM.pdf]

Supporting Information

Testing the impact of effective population size on speciation rates – a negative correlation or lack thereof in lichenized fungi

Jen-Pan Huang[1]*, Steven D. Leavitt[2], and H. Thorsten Lumbsch[1]
[1]Integrative Research Center, The Field Museum
[2]Department of Biology & M. L. Bean Museum, Brigham Young University

*e-mail: jhuang@fieldmuseum.org

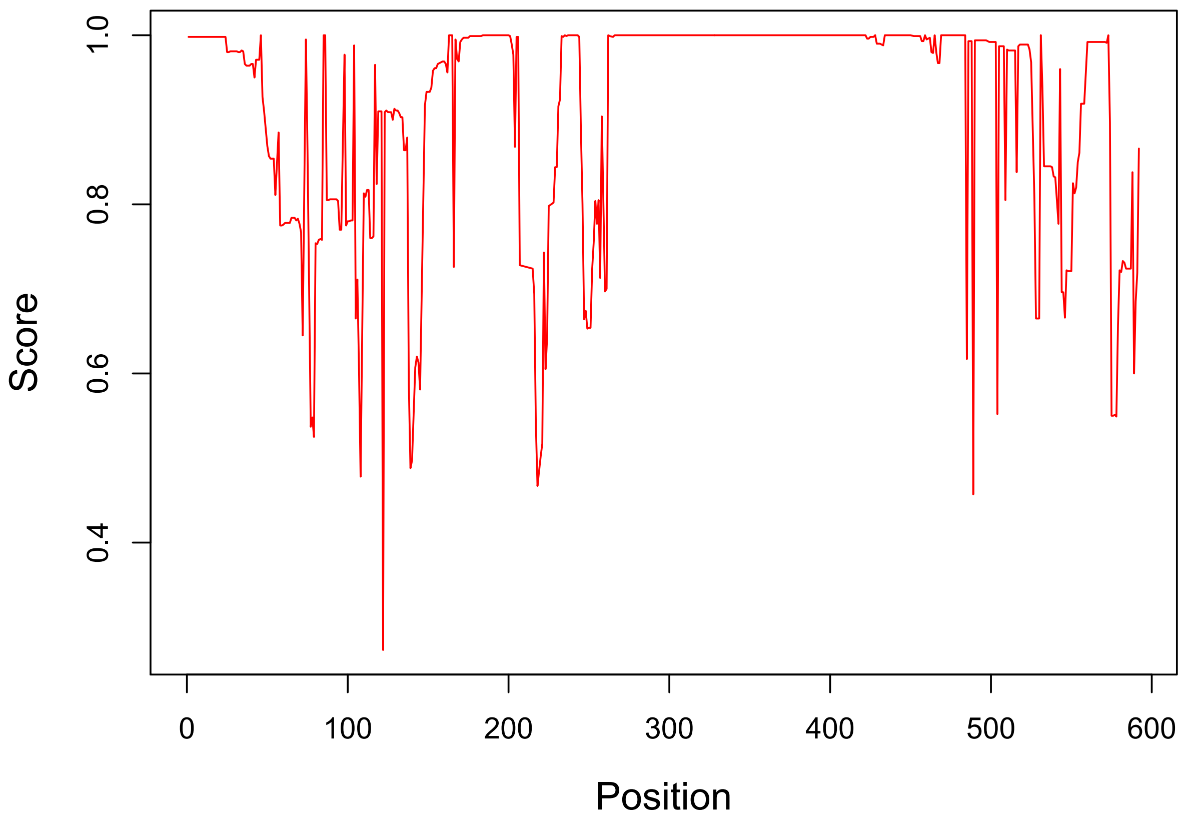

Fig. S1 – Guidance 2 scores for each site of the MAFFT alignment result.

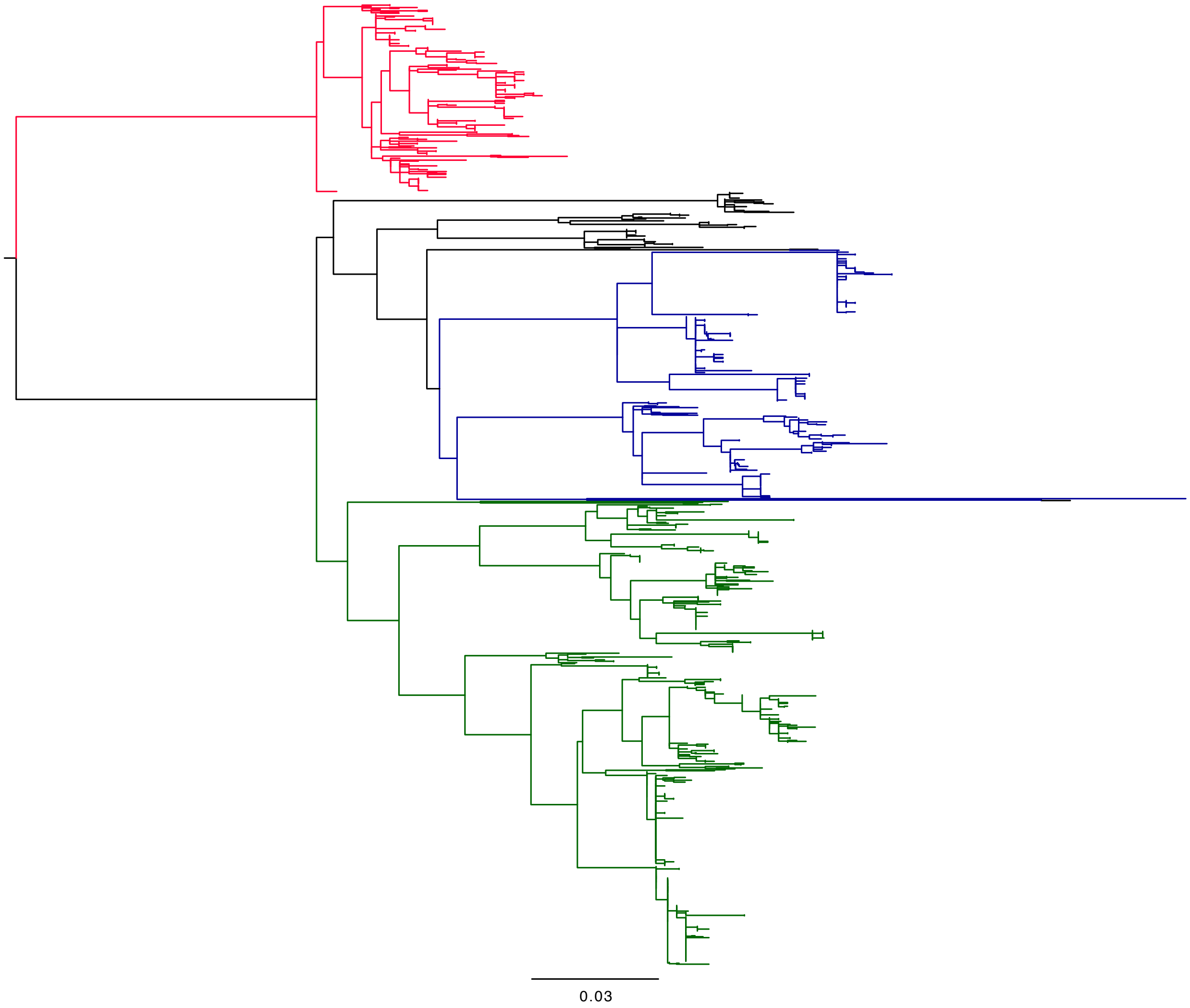

Fig. S2 – A ML phylogeny using ITS sequence data of the focal samples reconstructed using PhyML. Red clade: *Xanthoparmelia*; blue clade: *Melanelixia*; green clade: *Melanohalea*.

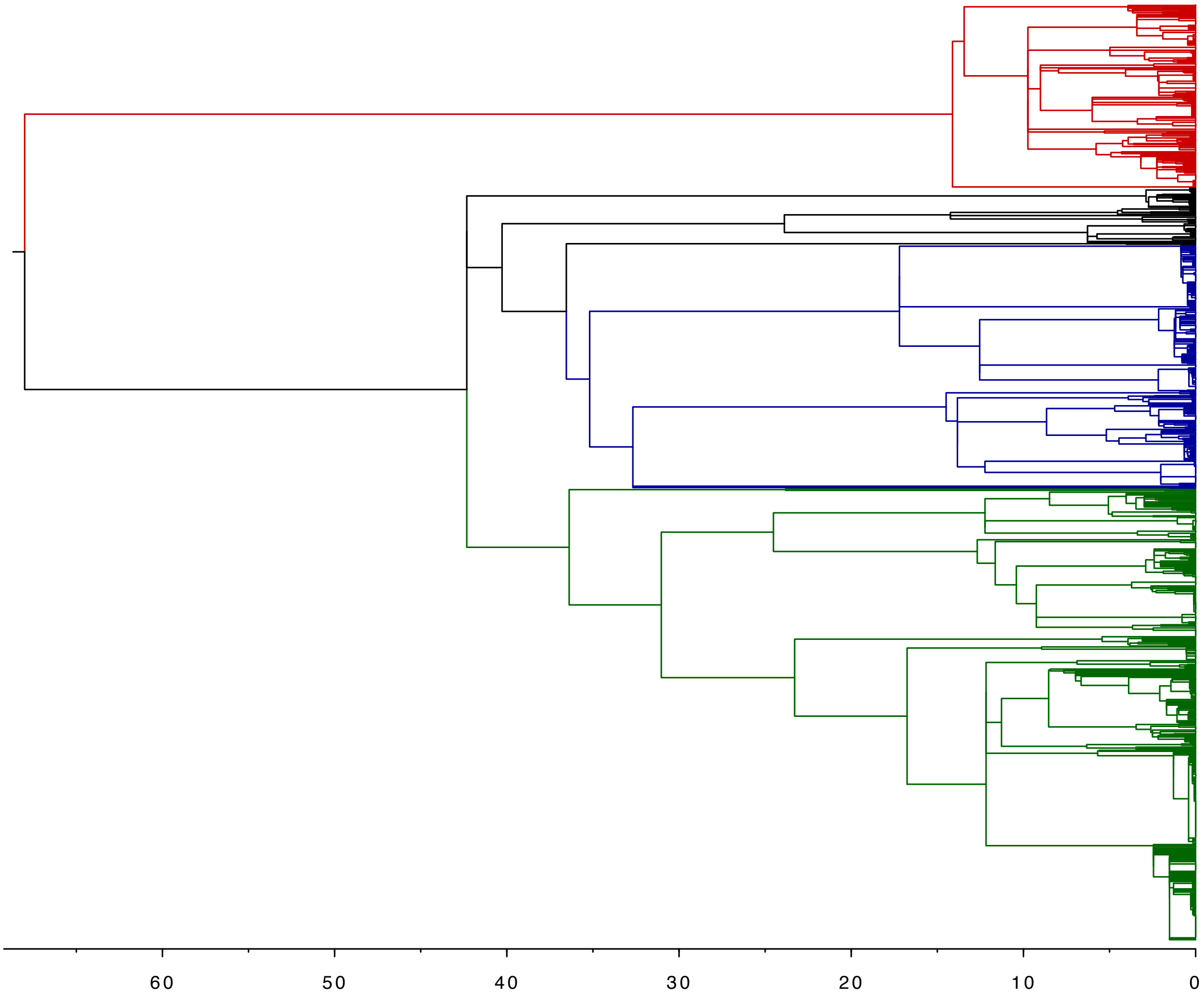

Fig. S3 – A time calibrated ultrametric tree of the sampled lichenized fungal ITS1 sequences. The scale is in million years. Red clade: *Xanthoparmelia*; blue clade: *Melanelixia*; green clade: *Melanohalea*.

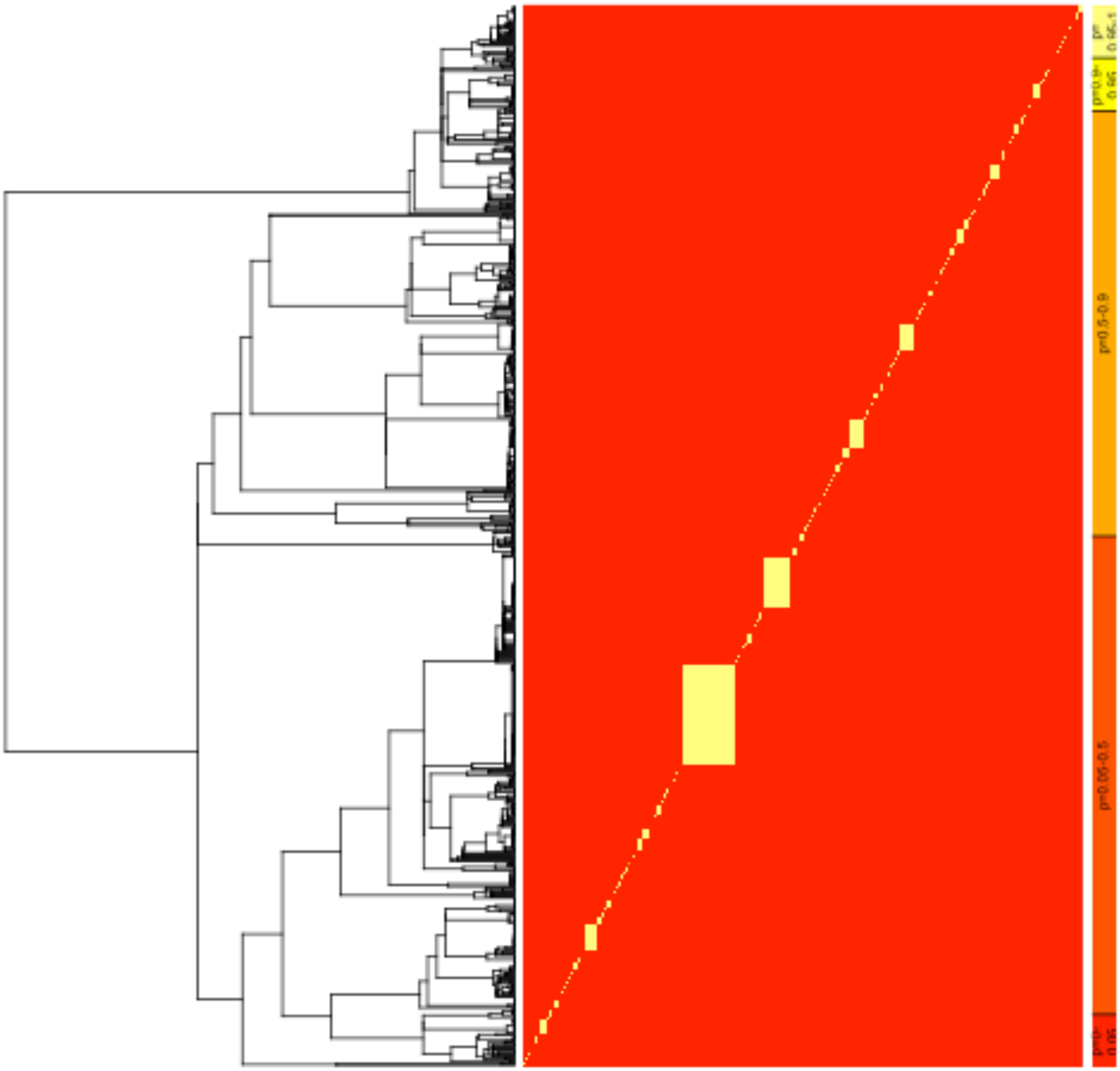

Fig. S4 – Species delimitation result from a bGMYC analysis. Colors in the heat map depicts the posterior probabilities of different individuals being designated as the same species.

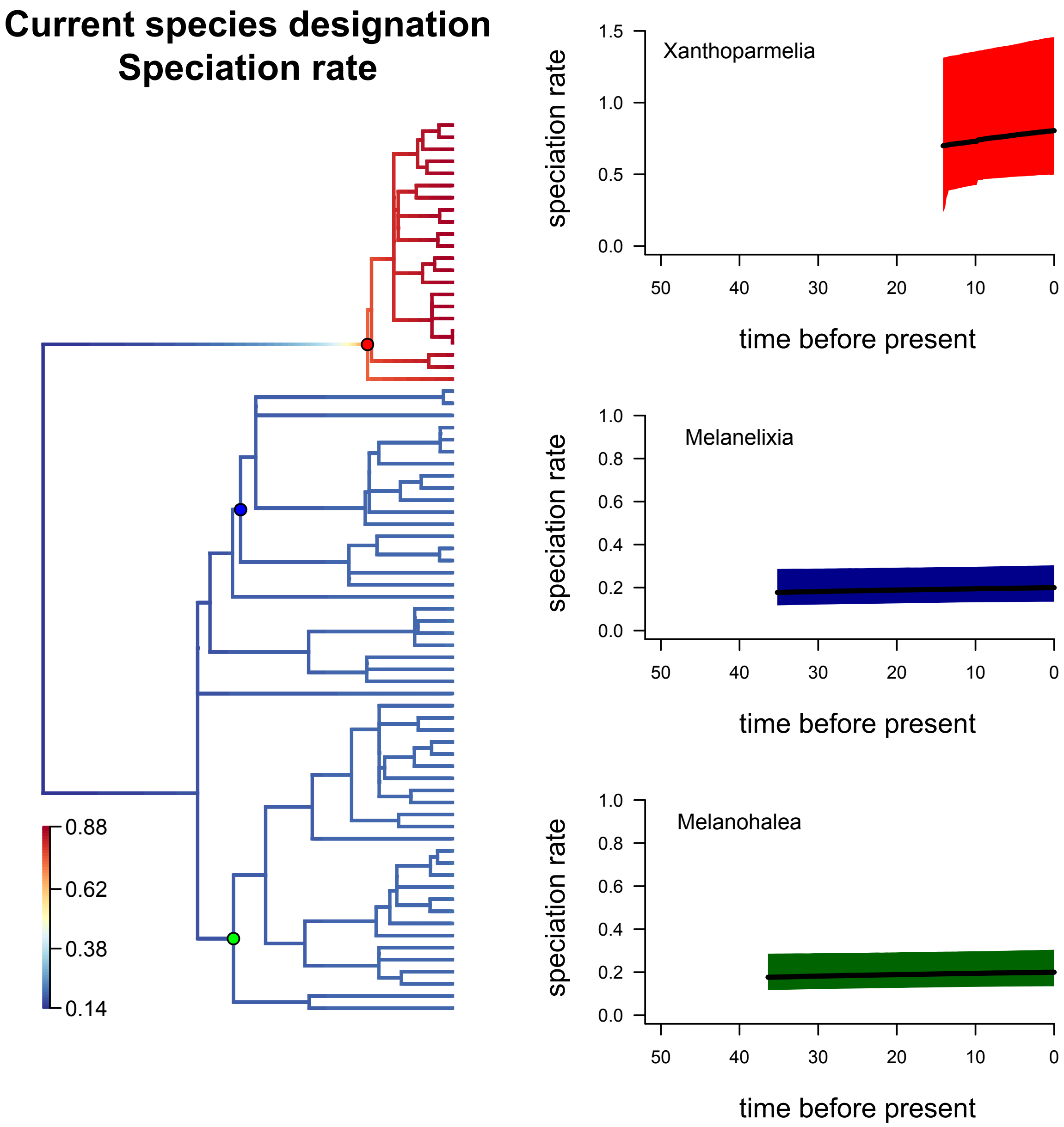

Fig. S5 – BAMM results based on species delineated using current taxonomic treatment. A BAMM plot showing changes in speciation rate along the reconstructed tree is shown in the left panel. Colored circles on nodes indicate the three focal clades of this study. A scale bar denotes color scheme utilized to represent the estimated speciation rate on nodes and branches. Calculated rate through time plots for three lichenized fungal clades are shown in the right panels. X-axes are in a scale of million years.

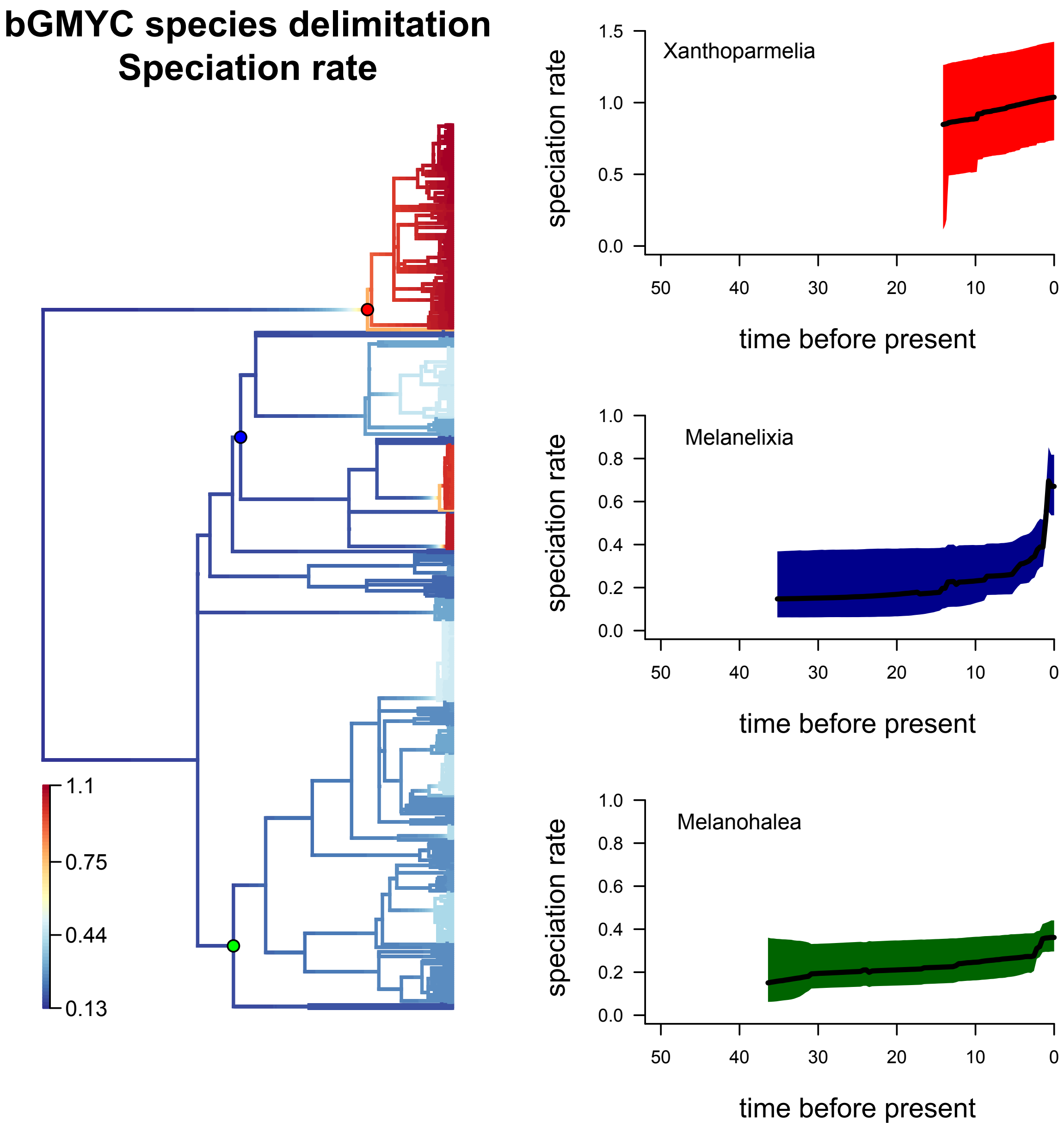

Fig. S6 – BAMM results based on species delineated using bGMYC. A BAMM plot showing changes in speciation rate along the reconstructed tree is shown in the left panel. A scale bar denotes color scheme utilized to represent the estimated speciation rate on nodes and branches. Colored circles on nodes indicate the three focal clades of this study. Calculated rate through time plots for three lichenized fungal clades are shown in the right panels. X-axes are in a scale of million years.

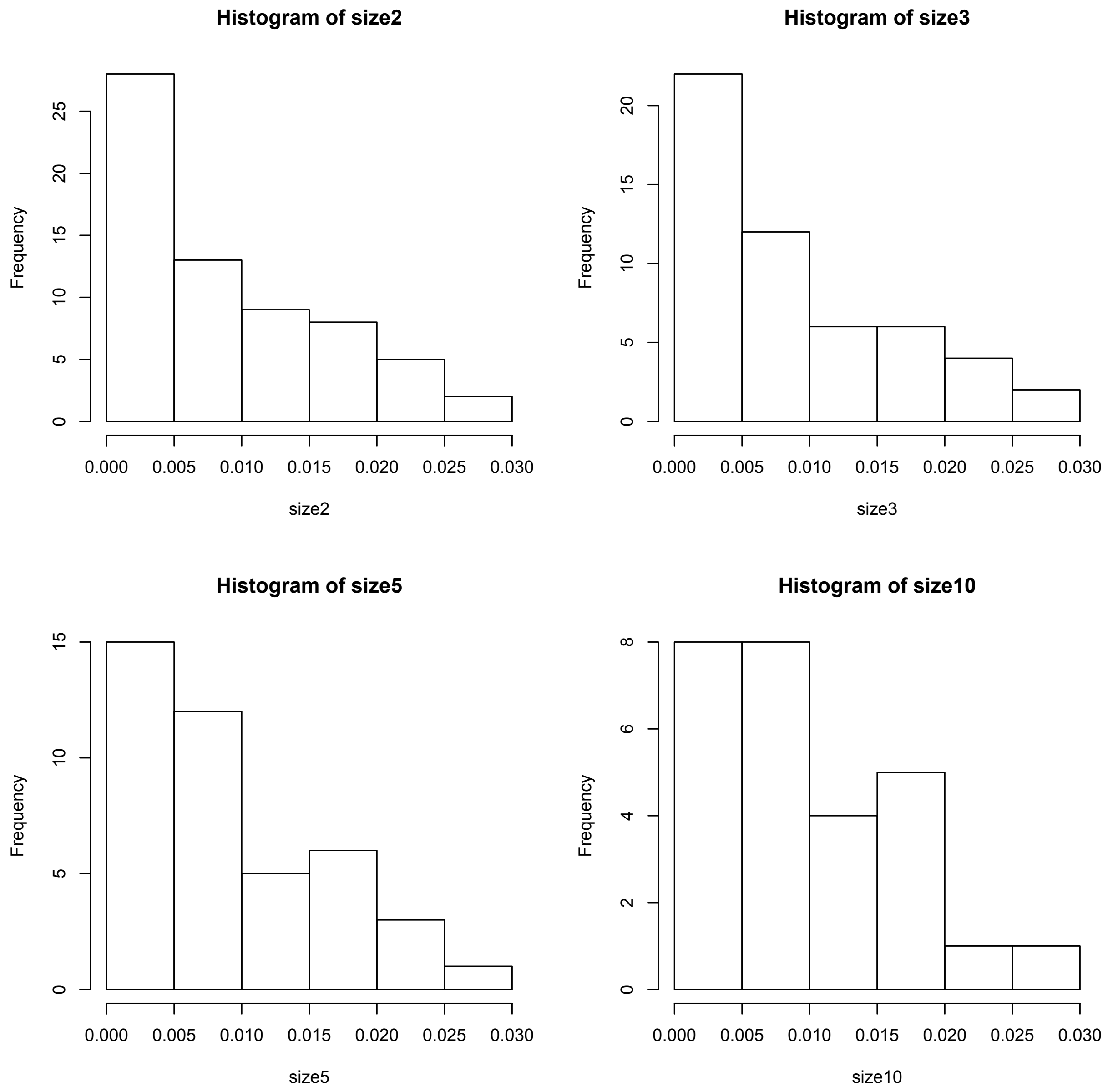

Fig. S7 – Frequency distributions of estimated population size (x-axes: theta calculated based on number of segregating sites). Different numbers of minimum individuals for species to be used in the calculations were applied (at least 2, 3, 5, and 10 individuals had to be sampled to be included in the analyses [size2, size3, size5, and size10, respectively]). Species here are delineated based on TN.

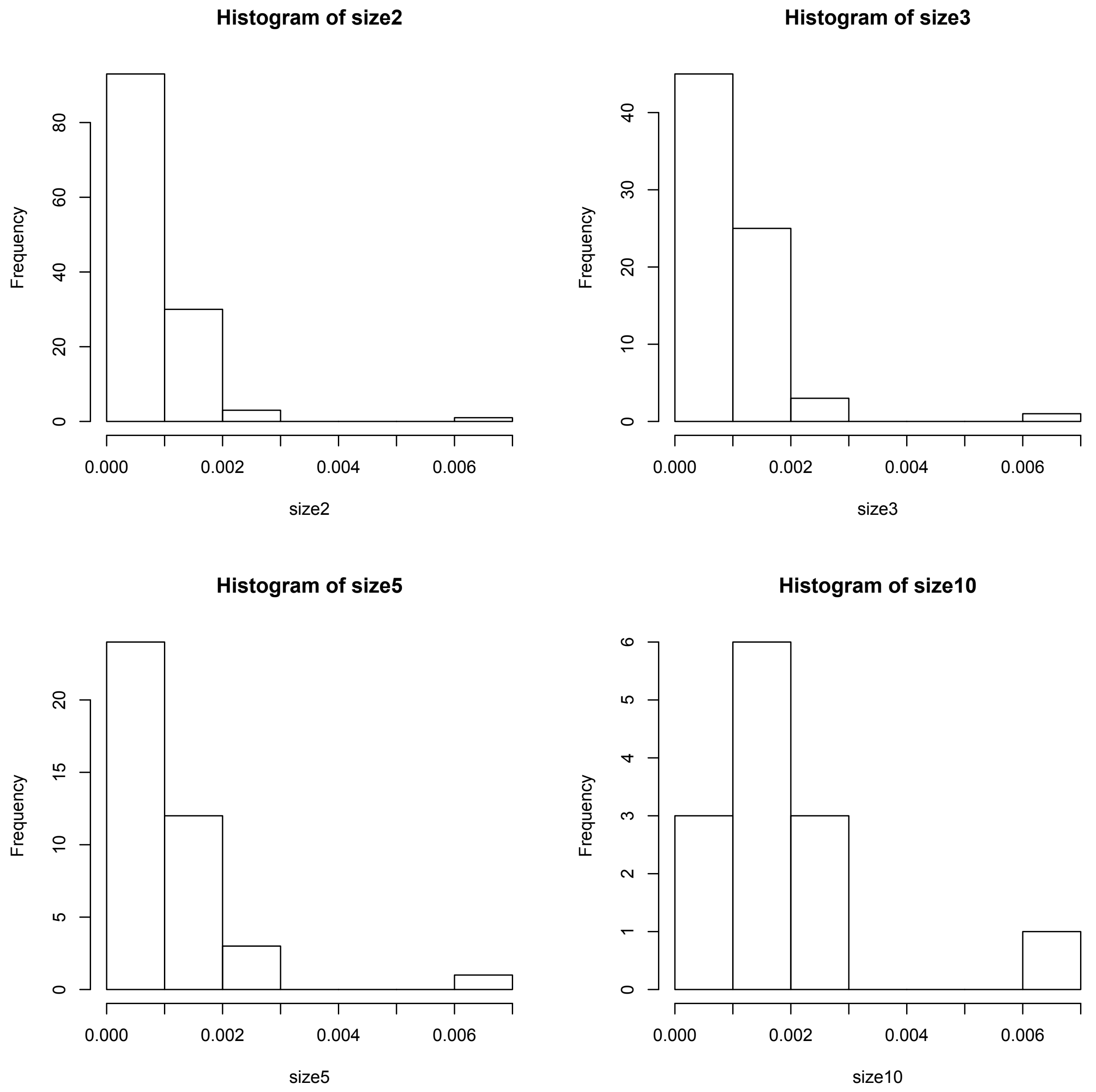

Fig. S8 – Frequency distributions of estimated population size (x-axes: theta values calculated based on number of segregating sites). Different numbers of minimum individuals for species to be used in the calculations were applied (at least 2, 3, 5, and 10 individuals had to be sampled to be included in the analyses [size2, size3, size5, and size10, respectively]). Species here are delineated based on bGMYC result.
